# Investigating portable fluorescent microscopy (CyScope^®^) as an alternative rapid diagnostic test for malaria in children and women of child-bearing age

**DOI:** 10.1186/1475-2875-9-245

**Published:** 2010-08-27

**Authors:** José Carlos Sousa-Figueiredo, David Oguttu, Moses Adriko, Fred Besigye, Andrina Nankasi, Moses Arinaitwe, Annet Namukuta, Martha Betson, Narcis B Kabatereine, J Russell Stothard

**Affiliations:** 1 WHO Collaborating Centre Schistosomiasis, Wolfson Wellcome Biomedical Laboratories, Department of Zoology, Natural History Museum, London, SW7 5BD, UK; 2 Department of Infectious and Tropical Diseases, London School of Hygiene and Tropical Medicine, Keppel Street, London WC1E 7HT, UK; 3 Vector Control Division, Ministry of Health, P.O. Box 1661, Kampala, Uganda

## Abstract

**Background:**

Prompt and correct diagnosis of malaria is crucial for accurate epidemiological assessment and better case management, and while the gold standard of light microscopy is often available, it requires both expertise and time. Portable fluorescent microscopy using the CyScope^® ^offers a potentially quicker, easier and more field-applicable alternative. This article reports on the strengths, limitations of this methodology and its diagnostic performance in cross-sectional surveys on young children and women of child-bearing age.

**Methods:**

552 adults (99% women of child-bearing age) and 980 children (99% ≤ 5 years of age) from rural and peri-urban regions of Ugandan were examined for malaria using light microscopy (Giemsa-stain), a lateral-flow test (Paracheck-Pf^®^) and the CyScope^®^. Results from the surveys were used to calculate diagnostic performance (sensitivity and specificity) as well as to perform a receiver operating characteristics (ROC) analyses, using light microscopy as the gold-standard.

**Results:**

Fluorescent microscopy (qualitative reads) showed reduced specificity (<40%), resulting in higher community prevalence levels than those reported by light microscopy, particularly in adults (+180% in adults and +20% in children). Diagnostic sensitivity was 92.1% in adults and 86.7% in children, with an area under the ROC curve of 0.63. Importantly, optimum performance was achieved for higher parasitaemia (>400 parasites/μL blood): sensitivity of 64.2% and specificity of 86.0%. Overall, the diagnostic performance of the CyScope was found inferior to that of Paracheck-Pf^®^.

**Discussion:**

Fluorescent microscopy using the CyScope^® ^is certainly a field-applicable and relatively affordable solution for malaria diagnoses especially in areas where electrical supplies may be lacking. While it is unlikely to miss higher parasitaemia, its application in cross-sectional community-based studies leads to many false positives (i.e. small fluorescent bodies of presently unknown origin mistaken as malaria parasites). Without recourse to other technologies, arbitration of these false positives is presently equivocal, which could ultimately lead to over-treatment; something that should be further explored in future investigations if the CyScope^® ^is to be more widely implemented.

## Background

With more than one million people dying every year from malaria, prompt and correct diagnosis leading to accurate epidemiological assessments and better case management is crucial. This is particularly important as fears of emergence of parasite drug resistance to the recently introduced artemisinin-based combination therapy (ACT) are increasing [1]. The balance between unnecessary use of ACT and correct case management must become the priority for Roll-Back Malaria programmes in endemic countries. The WHO recommends malaria case management, where possible, to be based on parasitological diagnosis, except when considering young children in areas of high transmission where lack of resources or urgency of response temporarily limits its application [2]. However, most malaria cases in deprived areas tend to go undiagnosed and, more often then not, untreated, as clinical diagnosis has limited specificity [3] and quality malaria microscopy is difficult to implement at rural clinic levels.

The expansion of parasitological diagnosis leading to better case management even in the most remote areas endemic for malaria will soon rely predominantly on rapid diagnostic tests (RDTs) [4], and until recently they all consisted of lateral-flow immunochromatographic devices that detect parasite-specific antigens in the blood (for review on applicability and performance of a selection see [5,6]). More recently, however, a new type of RDT has been developed using fluorescent microscopy: the portable, battery-operated CyScope^® ^(Partec, Germany) [7], which aims at reducing time and training needed for diagnosis (for information on technology see [8]). Albeit classifiable as an RDT (average time per diagnosis under 10 min), the CyScope^® ^is also thought able to quantify infection parasitaemia, by counting the number of malaria parasites per white blood cells, a feature missed by all lateral-flow tests, as well as, being used for direct morphological inspection of red blood cells. Understanding parasitaemia levels pre- and post-treatment is crucial particularly for in-patient case management in health centres, clinics or hospital wards. Recently, a pioneering cross-sectional facility-based analytical study of the diagnostic performance of the CyScope^® ^was conducted in Sudan with very promising results: sensitivity of 98.2% and specificity of 98.3% (gold standard: light microscopy) was obtained [9].

The affordable pricing, portability and compact design of the CyScope^®^, and the fact that reagents do not require cold storage, make the method a potentially attractive alternative for malaria diagnosis in the rural setting.

This article reports on the diagnostic performance of the CyScope^®^, as well as the lateral-flow Paracheck-Pf^® ^test (Orchid, India), as RDTs for malaria in children under five years of age and women of child-bearing age during cross-sectional epidemiological surveys in rural and semi-urban environments in Uganda.

## Methods

### Study sites and population

The study was conducted between October and November 2009 in six rural villages of Uganda where malaria is particularly problematic and responsible for considerable infant mortality: Bugoigo (1°54'01''N, 31°24'21''E), Walukuba (1°50'14''N, 31°22'56''E) and Piida (1°49'22''N, 31°19'19''E) located along the shoreline of Lake Albert in Buliisa District, Bugoto (0°19'12''N, 33°37'43''E), Bukoba (0°18'43''N, 33°29'33''E) and Lwanika (0°21'12''N, 33°26'40''E) located along the shoreline of Lake Victoria in Mayuge District. To make an assessment in a more urban setting, the town of Kasangati (0°26'07''N, 32°36'03''E) was chosen (suburb to Uganda's capital, Kampala). Individuals from Buliisa and Mayuge districts were inspected for malaria as part of a large-scale cohort study investigating maternal and child health in rural Uganda (the schistosomiasis in mothers and infants - SIMI project funded by the Wellcome Trust). This survey, using the CyScope^®^, constituted the 6-month follow-up of the SIMI cohort (baseline surveys took place in April-June 2009). Recruitment at baseline involved a community-wide sensitization (door-to-door); those willing to participate were instructed report to a specific location within the village (health centre, church or school) the following day. SIMI's recruitment criterion at baseline was to include any mother aged 15 to 55 years of age accompanied by at least one child. Exclusion from the cohort study would occur if mother and child(ren) failed to provide faecal samples for schistosomiasis diagnosis. Individuals from Kasangati town were investigated solely for malaria (i.e. conducting a cross-sectional survey from the local health centre). The total study population included 552 adults (244 mothers from Buliisa District, 241 mothers from Mayuge District and 67 adults from Kasangati town) and their 980 children (414 from Buliisa District, 486 from Mayuge District and 80 from Kasangati town). All adults gave written informed consent on their behalf, and on behalf of their children. Malaria treatment - ACT (20 mg arthemether/120 mg lumefantrine) for all except pregnant women (oral quinine) - was offered on the basis of a positive Giemsa blood film and/or positive Paracheck-Pf^® ^test in an outpatient setting.

### On-site malaria diagnoses

All individuals (552 adults and 980 children) were finger-pricked for preparation of thick and thin blood smears, and the latter was fixed in absolute methanol for 30 seconds. The slide was then immersed in a freshly prepared 10% pre-filtered Giemsa stain solution for 5-10 minutes, washed with water and left to dry. Asexual parasites and gametocytes were counted against 200-500 leucocytes and converted to number of parasites per volume assuming 8,000 leucocytes/μL of blood. Although gametocytes are not normally included when estimating an individual's parasitaemia, they were included for this methodology to allow comparison between the numbers of parasitic forms identified by both light and fluorescent microscopy. Slides were considered negative when no parasites were detected after viewing 100 microscopic fields. A successful stain was achieved in 99% of slides (548 adults and 968 children). Reading took place under 1000 × magnifications using a light compound microscope in the field under oil-immersion and using a portable generator as local power supply. As a quality control cross-check, all slides were transported to Kampala and re-read at the Vector Control Division (Ugandan Ministry of Health) to ascertain field accuracy (3 slides from adults and 12 slides from children were broken during transportation). All light microscopy was performed by experienced Vector Control Division technicians.

Rapid testing for malaria was performed by fluorescent microscopy (CyScope^®^Malaria, Partec, Münster, Germany) and with a lateral-flow test (Paracheck-Pf^®^, Orchid Biomedical Systems, Goa, India).

Fluorescent microscopy was performed according to the manufacturer's indications [7]: the fresh blood sample was transferred onto the Partec Malaria Test Slide (ready-prepared containing the necessary lyophilized reagents including the DNA stain), and a cover glass directly placed on top. The slide was then viewed (maximum of four hours between preparation and diagnosis) using 400 × magnification and fluorescent light for rapid diagnosis (qualitative testing on 472 adults and 851 children) or using 1000 × magnification under oil-immersion and fluorescent light to ascertain levels of parasitaemia (quantitative testing on 238 adults and 386 children). In the latter, parasites (viewed as small circular fluorescent bodies) were counted against 200-500 leucocytes and converted to number of parasites per volume assuming 8,000 leucocytes/μL blood. Slides were considered negative when no parasites were detected after viewing 100 microscopic fields. 10% of low parasite density infections (<200 parasites per μL/blood) were photographed using the CyScope^® ^camera and kept by the lead researcher for future reference. The person operating the fluorescent microscope was unaware of corresponding light microscopy results as readings were undertaken in separate rooms. Said technician works for the Vector Control Division of Uganda's Ministry of Health and is highly experienced in malaria diagnosis using light microscopy.

Lateral-flow tests were completed on all individuals (552 adults and 980 children) with the same finger prick blood sample as the thick/thin smears and fluorescent microscopy. Test kits were stored as directed by the manufacturer and quality of package desiccant was checked before use. The fresh blood sample was transferred directly to the sample pad by the provided sample applicator. All tests were labelled with patient ID numbers and village and results were recorded 15 minutes after adding 6 drops (300 μL) of clearing buffer. Presence of both the control and test lines indicated a positive result for *Plasmodium falciparum *and presence of only the control line indicated a negative result. In any case where the control line did not appear, the result was considered invalid and the test was repeated. The person recording the RDT result was unaware of corresponding microscopy results.

### Diagnostic performance

Diagnostic performance of both fluorescent microscopy and the lateral-flow test as diagnostic tests for malaria was established using Giemsa-stained light microscopy (double reads) as gold-standard. Sensitivity (SS), specificity (SP), positive predictive value (PPV) and negative predictive value (NPV) were calculated [10]. The diagnostic performance of each test was calculated using children and adults as separate target populations. Diagnostic performance of fluorescent microscopy and the lateral-flow test was compared using the McNemar's χ^2 ^test [11].

The correlation between light microscopy slide reads performed in the field and back in Kampala was calculated as well as between light microscopy slide reads performed in the field and fluorescent microscopy slide reads. Additionally, and to ascertain not only the correlation but also the agreement between light and fluorescent microscopy, a Bland-Altman plot was performed. The Bland-Altman plot is a tool for comparing two different methods of measuring the same value, when the true value being measured is not known. The purpose of a Bland-Altman plot is to try and determine whether fluorescent microscopy is better than light microscopy, using a hypothesis testing approach [12]. In these analyses, slide reads by both methodologies were log transformed to approach normality.

Receiver operating characteristics (ROC) analyses were also performed [13-15], plotting the true positive rate (Sensitivity) as a function of the false positive rate (100-Specificity). For this analysis, data from adults and children were compiled into a single population in an attempt to maximize numbers. Calculation of standard error (SE) of area under the ROC curve was performed according to [16] and 95% confidence intervals were estimated as described below.

### Data analysis

Data were collected from each individual using pro-forma data sheets, which were then entered using Microsoft Excel™. The data thus collated were analysed using R statistical package^® ^v 2.8.1. For prevalence values and the various point estimates of diagnostic precision, 95% confidence intervals (CI_95_) were estimated using the binomial exact method [17]. Prevalence comparisons were performed using (one-tailed) Fisher's exact modification of the 2 × 2 chi-squared test [18].

### Ethical review

Ethical permission for the study was granted by the London School of Hygiene and Tropical Medicine (LSHTM 5538.09) and the National Council of Science and Technology, Kampala, Uganda.

## Results

The number of individuals included in the study was 552 adults (99% were women, 18% of which were pregnant, aged between 15 and 54, mean age 26) and 980 children (female to male ratio of 1.1, 99% aged between 5 months old and 5 years old). Fever, defined as under-arm temperature higher then 37.5°C, was diagnosed in 2.6% of adults and 6.6% of children, and only five children reported with symptoms of complicated malaria detected visually (jaundice, impairment of consciousness, weakness or severe anaemia). Light microscopy identified malaria prevalence levels reaching 24.6% (CI_95 _21.1 - 28.5%) in adults and 71.7% (CI_95 _68.7 - 74.5%) in children. Three infections were identified as non-*P. falciparum *malaria, likely *Plasmodium malariae*. The mean parasite count in all positive slides was 1464 per μL of blood (median 520, maximum 38 × 10^3^) for adults and 9928 per μL of blood (median 1080, maximum 55 × 10^4^) for children. Fluorescent microscopy identified malaria prevalence levels reaching 68.0% (CI_95 _63.6 - 72.2%) in adults and 86.5% (CI_95 _84.0 - 88.7%) in children. The mean parasite count in all positive slides was 1149 (median 120, maximum 32 × 10^3^) in adults and 5611 (median 1000, maximum 64 × 10^3^) in children. The lateral-flow rapid diagnostic test identified malaria prevalence levels reaching 24.1% (CI_95 _20.6 - 27.9%) in adults and 68.0% (CI_95 _63.6 - 72.2%) in children.

Malaria prevalence levels according to both light microscopy and the lateral-flow RDT were significantly lower in children from the Kasangati town than in children living in shoreline villages from Buliisa and Mayuge districts (*P *< 0.0001). This was not observed in adults using any diagnostic test, or in children, using fluorescent microscopy. See Additional File 1 for prevalence (and CI_95_) and mean parasitaemia in adults and children detailed by location.

**Figure 1:**
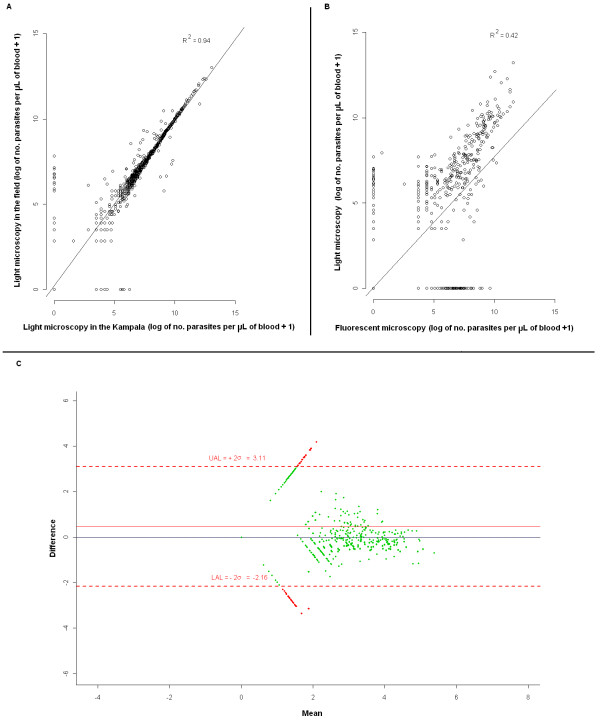
**Graphical representation of correlation between: A) Giemsa-stained slide reads using light microscopy in the field and in Kampala using Ministry of Health facilities and between B) slides read using fluorescent microscopy (CyScope^®^) and Giemsa-stained slide reads using light microscopy in the field**. C) Bland-Altman difference plot comparing fluorescent and light microscopy in the field. The points are plotted with the difference between fluorescent and light microscopy on the y-axis, and the mean of the two observations on the x-axis. The confidence bounds are plotted as dotted red lines, and all points within the confidence bounds are coloured green, all points outside the confidence bounds in red. The region of agreement is the area within the confidence bounds. The black solid line is the "difference = zero line" and the red solid line indicates the "average difference".

Importantly, there was a very good correlation (R^2 ^= 0.94) between light microscopy reads performed in the field and those performed in Kampala (see Figure 1A). However, a much poorer correlation was found (R^2 ^= 0.42) between reads by light and fluorescent microscopy (Figure 1B). The Bland-Altman plot illustrates the lack of agreement between methodologies particularly for cases diagnosed as negative for malaria by either (but not both) methodologies (Figure 1C). For positive parasitaemia the level of agreement remains within the confidence bounds.

### Diagnostic performance of CyScope^® ^and Paracheck-Pf^®^

Fluorescent microscopy (CyScope^®^) was found to be sensitive (92.1% in adults and 86.7% in children) for diagnosing malaria infections; however, with low specificity, particularly in children (38.8% in adults and 28.6% in children). The lateral-flow RDT (Paracheck-Pf^®^) significantly out-performed fluorescent microscopy in mothers (McNemar's χ^2 ^175.1245, *P *< 0.0001) and children (McNemar's χ^2 ^66.3594, *P *< 0.0001), with high sensitivity (91.9% in adults and 98.4% in children) and specificity (98.1% in adults and 96.2% in children) at diagnosing malaria infections. See Table 1 for all results and confidence intervals.

**Table 1:** Diagnostic performance of fluorescent microscopy (CyScope^®^) and the lateral-flow reagent strip (Paracheck-Pf^®^) using light microscopy (double reads) as gold-standard.

		**FLUORESCENT MICROSCOPY V. LIGHT MICROSCOPY**	**PARACHECK-PF V. LIGHT MICROSCOPY**
		**468 ADULTS AND 836 CHILDREN**	**546 ADULTS AND 951 CHILDREN**
Adults	Sensitivity (%)	86.7 (79.3 - 92.2)	91.9 (85.9 - 95.9)
	Specificity (%)	38.8 (33.6 - 44.1)	98.1 (96.2 - 99.2)
	PPV (%)	32.8 (27.7 - 38.3)	93.9 (88.4 - 97.3)
	NPV (%)	89.4 (83.4 - 93.8)	97.3 (95.3 - 98.7)
	
Children	Sensitivity (%)	92.1 (89.6 - 94.1)	98.4 (97.2 - 99.2)
	Specificity (%)	28.6 (22.8 - 34.9)	96.2 (93.1 - 98.2)
	PPV (%)	77.1 (73.9 - 80.2)	98.5 (97.3 - 99.3)
	NPV (%)	57.9 (48.3 - 67.1)	95.8 (92.7 - 97.9)

Values are expressed in % and brackets indicate CI_95_; PPV stands for positive predictive value, NPV stands for negative predictive value

Using fluorescent microscopy as a quantitative diagnostic test (parasitaemia counts) using data from 620 individuals (prevalence by light microscopy of 54.0% in this population), the resulting area under the ROC curve was 0.8059 (CI_95 _0.77205 - 0.83968, SE 0.0173), with a reported sensitivity of 89.6% and specificity of 37.2%, where 65.5% of diagnoses correctly classified. Interestingly, the ROC analysis demonstrated that the parasitaemia threshold for optimum performance by fluorescent microscopy is 400 parasites per μL of blood: sensitivity of 64.2% and specificity of 86.0%, with 74.2% of diagnoses correctly classified.

## Discussion

The cohort selection in the rural villages around Lakes Albert (Buliisa district) and Victoria (Mayuge district) was targeted at women of child-bearing age and preschool-aged children (≤5 years of age) where malaria is known to be problematic and co-infection with intestinal schistosomiasis commonly occurs. Although most mothers were younger than 45 years of age, some older guardians were recruited in an attempted to maximise numbers, which explains ages reaching 54 years. The survey in Kasangati town was staged in the local health centre, and people were recruited from the attending line (these patients were complaining of several symptoms/pathologies, many of which unrelated to malaria). Here, mothers with their young children were recruited; however, some fathers also wished to participate, and to increase sample sizes were included in the study. This explains why 1% of our sample of adults was male, and why 1% of our children were older than 5 years of age (maximum age recorded was 8 years).

Levels of malaria in young children according to light microscopy were high, particularly in both rural settings (76.1% in Buliisa district and 72.9% in Mayuge district); nevertheless, children from Kasangati town, a far more urbanized settlement, were still highly affected by malaria, with prevalence reaching 47.5%. Mothers were far less affected by malaria, with levels varying only slightly from 20.9% (Kasangati town) to 26.4% (Buliisa district). 21% of the pregnant mothers were infected with malaria. These levels of malaria are of particular concern as many of the children and mothers positive at the time of this survey (Buliisa and Mayuge districts) had been diagnosed and treated (20 mg arthemether/120 mg lumefantrine - children and non-pregnant mothers) in May-June (baseline survey) and September 2009 (3-month follow-up), indicating very high re-infection rates in these rural environments. In fact, previous observations on entomological inoculation rates suggest that individuals in rural Uganda can receive >1,500 infective bites per year (*Anopheles gambiae s.s*. and *Anopheles funestus*) [19].

The great majority of infections were *P. falciparum*, according to light microscopy. In corroboration, additional data from real-time polymerase chain reaction analysis from blood-spots collected at the baseline survey (cohort establishment six months prior to these surveys) reveals prevalence levels of non-*P. falciparum *malaria varying from 3.0% in Bugoigo village, Bullisa District (1.5% *P. malariae *and 1.5% *Plasmodium ovale*) to 17% in Bukoba village, Mayuge district (11% *P. malariae *and 6% *P. ovale*) (unpublished). Prevalence levels and proportion of the different *Plasmodium spp*. reported in this article are within previously reported ranges [19-23].

The lateral-flow rapid diagnostic test Paracheck-Pf^® ^performed well in field conditions (91.9% and 98.4% sensitivity and 98.1% and 96.2% specificity, in adults and children respectively, rate of invalid tests <0.01%). These values also fall within previously reported ranges [5,6] and further suggest that although non-*P.f*. infection may occur in the study areas, a vast proportion occur in co-infection with *P. falciparum*.

### Fluorescent microscopy by CyScope^®^

**Figure 2:**
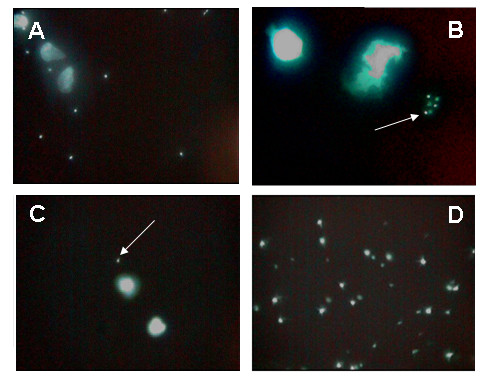
**Photographs taken using the CyScope^® ^camera (× 1000 magnification) illustrating**: A) clear case of parasitaemia with parasites fluorescing around white blood cells; B) non-*P. falciparum *malaria parasites (arrow), confirmed by positive light microscopy and negative result on Paracheck-Pf^®^; C) case of fluorescent body resembling a malaria parasite (arrow) next to two white blood slides (this case was negative under light microscopy and by Paracheck-Pf^®^); and D) debris resulting from disintegrated white blood cell, some resembling in size and shape a malaria parasite.

Malaria diagnosis using fluorescent microscopy by portable CyScope^® ^is fairly simple, relatively cheap (£818 for the microscope and £0.40 per test) and was found to be easy to apply even in field conditions, and when parasitaemia is above 400 parasites per μL of blood, positive cases are easy to identify (Figure 2A). Additionally, this new technology allows for both qualitative and quantitative diagnoses, important for rapid diagnoses and long-term case management (regression of parasitaemia during treatment), respectively. Although unable to differentiate between *Plasmodium *spp. (no visual distinction in fluorescence size, shape or intensity), all *Plasmodium *spp. fluoresce using this methodology (Figure 2B); crucial in areas where non-*P.f*. infections are common.

In practice, unequivocal diagnosis of malaria infection using fluorescent microscopy proved to be unreliable at the community and individual levels, particularly in populations where the majority of malaria cases are afebrile and uncomplicated, and in meso-endemic areas such as Kasangati town. Here, the prevalence of malaria in children according to fluorescent microscopy was close to 80% higher than that reported by light microscopy (compared to +20% for Buliisa and +14% for Mayuge). This excessive diagnosis of malaria positives was repeated in the adults, only this time in all locations surveyed (+190% for Kasangati, +170% for Mayuge and +180% for Buliisa). Importantly, even in hyper-endemic rural environments, this methodology was shown to be significantly less reliable than the already established, cheap and rapid lateral-flow test: the Paracheck-Pf^®^.

The use of fluorescent microscopy was characterised by the rate at which false positives were being diagnosed, potentially leading to drug waste (specificity levels of 38.8% in mothers and 28.6% in children). Many false positives were diagnosed with extremely low parasitaemia (40 parasites per μL of blood using the CyScope^®^) with only very few parasite-like fluorescent bodies found in the whole slide (see Figure 2C for an example). Although of unknown source, the high presence of false positives should be further investigated; one potential confounder could be co-infection with schistosomes. As these worms reside directly in the blood stream and daily regurgitate their blood meals sloughing intestinal cells into the host blood stream, such cellular contents likely contain broken down nuclei which might give rise to fluorescing bodies viewed by CyScope^®^. In addition, field studies, unlike carefully controlled laboratory/hospital/clinic-based studies, are vulnerable to different elements, and unidentified DNA-containing bodies or dust particles were found to cause problems at certain times. Interestingly a recent SIMI survey using the CyScope^® ^has established that a transient bacteraemia or bacteria/yeasts contaminants from the area where the finger-prick can be mistaken for a malarial infection. Additionally, on warmer survey days, leaving CyScope^® ^blood slides at ambient temperatures for a few hours was found to lead to quick disintegration of white blood cells (whether due to drying or bleaching by UV is still to be investigated), whose fragments could easily be mistaken for parasites (Figure 2D); unlike Giemsa-stained slides, CyScope^® ^slides cannot be kept as an archive.

Sensitivity levels were acceptable, reaching 86.7% in mothers and 92.1% in children, but lower than previously reported values for this methodology [9] (albeit under very different survey settings and study populations), and under the 95% threshold required in a malaria rapid diagnostic test [24]. Majority of false negatives diagnosed by fluorescent microscopy were of low parasitaemia according to light microscopy (≤400 parasites per μL of blood).

## Conclusions

Fluorescent microscopy by CyScope^® ^is a field-applicable and affordable solution to malaria diagnoses in the rural setting. This methodology showed promise at identifying malaria positives (good sensitivity); however, high rate of false positives was surprising and should be more carefully examined, as this can lead to ineffective treatment streamlining and drug waste. Further studies are needed to determine the cost-effectiveness and longer term performance of the CyScope^® ^in diagnosing malaria, especially if this methodology is to scaled-up in the rural health care setting.

## Competing interests

The authors declare that they have no competing interests.

## Authors' contributions

JRS, NBK, MB and JCSF conceived and participated in the design of the study. JRS, MB, DO and JCSF coordinated the study. JCSF performed statistical analysis. DO and MAd performed fluorescent microscopy reads, FB and MAr performed the lateral-flow diagnostic tests, ANam and ANan performed light microscopy both in the field and in Kampala. JCSF, JRS and MB helped to draft manuscript. All authors contributed towards the final manuscript.

## Supplementary Material

Additional file 1 **Supplemental table**. Prevalence (and CI_95_) and mean parasitaemia in adults and children detailed by location. Click here for file
