# Supplementary material for: Investigating portable fluorescent microscopy (CyScope®) as an alternative rapid diagnostic test for malaria in children and women of child-bearing age
Source: Malar J. 2010 Aug 27;9:245. doi: 10.1186/1475-2875-9-245 (PMC2940895; doi:10.1186/1475-2875-9-245)
Supplement: Additional file 1 — Supplemental table. Prevalence (and CI95) and mean parasitaemia in adults and children detailed by location. [file 1475-2875-9-245-S1.DOC]

**Additional file 1** – Prevalence (and CI_95_) according to all malaria diagnostic tests performed (light microscopy using normal compound microscope, fluorescent microscopy using the CyScope® and the lateral-flow test Paracheck-Pf®) for diagnosing adults and children, with observations on mean, median and maximum values (max.) of the distribution of parasitaemia of positive cases according to light and fluorescent microscopy. ^¥^ Giemsa-stained slides read twice

|  | |  | Adults | | |  | Children | | |
| --- | --- | --- | --- | --- | --- | --- | --- | --- | --- |
|  |  |  | Buliisa | mayuge | kasangati |  | Buliisa | mayuge | kasangati |
|  |  |  |  |  |  |  |  |  |  |
| Light microscopy^¥^ | Prevalence in % (CI_95_) |  | 26.4  (20.9 – 32.4) | 24.3  (19.0 – 30.2) | 20.9  (11.9 – 32.6) |  | 76.1  (71.5 – 80.2) | 72.9  (68.7 – 76.8) | 47.5  (36.2 – 59.0) |
|  | Mean parasitaemia per μL of blood (median/max.) |  | 810  (480 / 9.7x10^3^) | 1475  (620 / 18x10^3^) | 4177  (400 / 38x10^3^) |  | 7665  (1000 / 24x10^4^) | 7925  (1040 / 45x10^4^) | 46700  (10300 / 55x10^4^) |
|  |  |  |  |  |  |  |  |  |  |
| Fluorescent microscopy | Prevalence in % (CI_95_) |  | 72.9  (65.6 – 79.5) | 66.4  (60.0 – 72.4) | 61.2  (48.5 – 72.9) |  | 91.9  (88.1 – 94.7) | 83.4  (79.8 – 86.6) | 85.0  (75.2 – 92.0) |
|  | Mean parasitaemia per μL of blood (median/max.) |  | 225  (80 / 2.8x10^3^) | 2024  (940 / 9.2x10^3^) | 1653  (80 / 32x10^3^) |  | 3856  (660 / 64x10^3^) | 3983  (1680 / 39x10^3^) | 12180  (240 / 11x10^4^) |
| Paracheck | Prevalence in % (CI_95_) |  | 24.6  (19.3 – 30.5) | 24.9  (19.6 – 30.9) | 19.4  (10.8 – 30.9) |  | 76.1  (71.7 – 80.1) | 72.6  (68.4 – 76.6) | 47.5  (36.2 – 59.0) |
|  |  |  |  |  |  |  |  |  |  |
